# Supplementary material for: Rational molecular and device design enables organic solar cells approaching 20% efficiency
Source: Nat Commun. 2024 Feb 28;15:1830. doi: 10.1038/s41467-024-46022-3 (PMC10902355; doi:10.1038/s41467-024-46022-3)
Supplement: Supplementary file 1 — Supplementary Information [file 41467_2024_46022_MOESM1_ESM.pdf]

Supplementary Information

Rational molecular and device design enables organic solar cells approaching 20% efficiency

Jiehao Fu[1,#], Qianguang Yang[2,3,4,#], Peihao Huang[3,4], Sein Chung[5], Kilwon Cho[5], Zhipeng Kan[6], Heng Liu[7], Xinhui Lu[7], Yongwen Lang[1,8], Hanjian Lai[8], Feng He[8], Patrick W. K. Fong[1], Shirong Lu[2,*], Yang Yang[9], Zeyun Xiao[3,4,*], and Gang Li[1,*]

[1]Department of Electrical and Electronic Engineering, Research Institute for Smart Energy (RISE), Photonic Research Institute (PRI), The Hong Kong Polytechnic University, Hung Hom, Kowloon, Hong Kong 999077, P. R. China.

[2]School of Materials Science and Engineering, Taizhou University, Taizhou 318000, P. R. China.

[3]Thin-film Solar Cell Technology Research Center, Chongqing Institute of Green and Intelligent Technology, Chongqing School, University of Chinese Academy of Sciences, Chinese Academy of Sciences, Chongqing 400714, P. R. China.

[4]University of Chinese Academy of Sciences, Beijing 100049, P. R. China

[5]Department of Chemical Engineering, Pohang University of Science and Technology, Pohang 37673, South Korea.

[6]School of Physical Science & Technology, Guangxi University, Nanning 530004, China

[7]Department of Physics, The Chinese University of Hong Kong, Shatin, Hong Kong 999077, P. R. China.

[8]Shenzhen Grubbs Institute and Department of Chemistry, Southern University of Science and Technology, Shenzhen, 518055, P. R. China.

[9]Department of Materials Science and Engineering, University of California Los Angeles (UCLA), Los Angeles, CA90095, USA.

[#] These authors contributed equally

*Correspondences: S.L. (e-mail: lushirong@cigit.ac.cn); Z.X. (e-mail: xiao.z@cigit.ac.cn); G. L. (e-mail: gang.w.li@polyu.edu.hk).

Supplementary Methods

Synthesis of compounds o-IN-2Cl: As shown in Supplementary Fig. 1, thionyl chloride (4.4 mL, 60 mmol) was added to a solution of 2,3-dichlorobenzoic acid (5.73 g, 30 mmol) in $CHCl_3$ (30 mL) under the protection of N_2. Subsequently, DMF (0.5 mL) was added to initiate the reaction. The reaction was stirred at 65 °C for 1 hour, then the excess thionyl chloride was removed under reduced pressure, and 2,3-dichlorobenzoic chloride was used for the next step without further purification. A mixture of malonyl dichloride (5.6 mL, 60 mmol) and $AlCl_3$(6.00 g, 45 mmol) in dry dichloromethane (30 mL) solution was flushed with N_2 for ten minutes, then 2,3-dichlorobenzoic chloride was added and the reaction mixture was refluxed at 65 ℃ overnight. After cooling to room temperature, the mixture was poured into 2M oxalic acid aqueous solution slowly. Then, the pH of the solution was tuned to around 7 by using $NaHCO_3$ aqueous solution. The crude product was extracted from dichloromethane and then purified by silica gel chromatography using dichloromethane as the eluent to obtain a yellow solid o-IN-2Cl (4.67 g, yield 72.4%). ^{1}H NMR (400 MHz, chloroform-*d*). δ 7.88 (d, J = 8.1 Hz, 1H), 7.81 (d, J = 8.1 Hz, 1H), 3.29 (s, 2H).^{13}C NMR (101 MHz, $CDCl_3$) δ 194.75, 193.22, 143.08, 142.10, 139.83, 136.86, 130.16, 121.78, 77.36, 77.05, 76.73, 45.28. GCMS (ESI) m/z: $[M+H]^+$ Calcd for $C_9H_4Cl_2O_2$:214; Found 214.

Synthesis of compounds o-IC-2Cl: As shown in Supplementary Fig. 1, sodium acetate (0.57 g, 7.0 mmol) was added to a solution of malononitrile (0.61 g, 9.3 mmol) and o-IN-2Cl (1.00 g, 4.7 mmol) in 20 ml of ethanol. The mixture was stirred for 1 h at room

temperature. Then 10 mL of water was added into the mixture, then acidified to pH 1-2 by using HCl (2 M), and the mixture was filtered. Then purified by silica gel chromatography using dichloromethane as the eluent to obtain a yellow solid o-IC-2Cl (0.94 g, yield 77.1%). ^{1}H NMR (400 MHz, Chloroform-*d*).δ 8.55 (d, *J* = 8.5 Hz, 1H), 7.96 (d, *J* = 8.5 Hz, 1H), 3.83 (s, 2H).^{13}C NMR (101 MHz, $CDCl_3$) δ 190.78, 163.15, 142.41, 136.88, 124.32, 111.65, 77.34, 77.02, 76.70, 43.93. GCMS (ESI) m/z: $[M+H]^+$ Calcd for $C_{12}H_4Cl_2N_2O_2$:262; Found 262.

Synthesis of compound o-BTP-eC9: As shown in Supplementary Fig. 1, the compound BTP-eC9-CHO (108.4 mg, 0.1 mmol), o-IC-2Cl (105.2 mg, 0.4 mmol) and chloroform (10 mL) were added to a 25 mL round bottom flask. Then pyridine (0.5 mL) was added. The reaction mixture was stirred at room temperature (25-30℃) for 4 hours. Methanol was added, and the precipitate was collected by filtration to obtain the crude product. Using petroleum ether: Chloroform (1:1) as the eluent, the crude product was further chromatographed on a silica gel column to obtain the compound o-BTP-eC9 (151.5mg, yield 96.3%). ^{1}H NMR (400 MHz, Chloroform-*d*) δ 9.16 (s, 2H), 8.58 (d, *J* = 8.4 Hz, 2H), 7.77 (d, *J* = 8.4 Hz, 2H), 4.83 (q, *J* = 8.7, 8.1 Hz, 4H), 3.19 (t, *J* = 7.9 Hz, 4H), 2.16 (dd, *J* = 9.4, 4.0 Hz, 2H), 1.86 (p, *J* = 7.9 Hz, 4H), 1.49 (p, *J* = 7.0 Hz, 4H), 1.28 (dt, *J* = 8.9, 3.5 Hz, 22H), 1.17 – 0.85 (m, 32H), 0.78 – 0.56 (m, 18H). ^{13}C NMR (101 MHz, $CDCl_3$) δ 185.07, 157.97, 154.07, 147.50, 145.30, 140.24, 140.01, 137.74, 136.60, 135.72, 135.31, 134.42, 134.40, 133.54, 133.24, 131.19, 130.83, 123.69, 120.02, 115.30, 114.95, 113.58, 77.33, 77.01, 76.70, 68.11, 55.83, 39.32, 31.86, 31.52, 31.20, 30.59, 30.40, 29.82, 29.45, 29.37, 29.29, 28.15, 27.92, 25.47, 25.22, 22.87, 22.81, 22.65, 22.49, 22.46, 14.09, 13.96, 13.80, 13.75. MALDI-TOF MS (m/z) calculated for $C_{86}H_{94}Cl_4N_8O_2S_5$:1572.48279 $[M]^+$, found: 1572.50153.

Dielectric constant test. Dielectric constants of neat BTP-eC9 and o-BTP-eC9 films were measured with an electrochemical workstation (Autolab) by applying 20 mV AC modulation using a capacitor architecture of ITO/active layer/Ag at different frequencies from 1K Hz to 10M Hz. Between 10K Hz and 100K Hz, a flat capacitive

response with respect to frequency is obtained. Relative dielectric constant (ε_r) can be calculated according to:

$$\varepsilon_r = \frac{C_p * D}{A * \varepsilon_0} \qquad (1)$$

Where C_p is the measured capacitance; D is the thickness of film; A is the contact area and ε_0 is the permittivity of free space.

TPV and TPC measurements. TPV is tested under the open-circuit and 1 sun intensity background light condition to explore the photovoltage decay. The subsequent voltage decay is then recorded by the digital storage oscilloscope to directly monitor charge carrier recombination. The intensity of light is 230 μW/cm^2 and the wavelength of light is 520 nm. The light pulse is 10 ns. The normalized curves are easier to compare the decay time and the slow decline one is the one with a long lifetime. Using the same equipment, TPC is tested under the short-circuit condition to explore the time-dependent extraction of photogenerated charge carriers. The photovoltage and photocurrent decay kinetics of all devices follow a mono-exponential decay: $\delta V = A\exp(-t/\tau)$ where t is the time, and τ is the decay time. The fitted decay time would not be affected by the A value, thus the TPV and TPC curves are normalized.

SCLC mobility measurements. Electron-only devices with the structure of ITO/ZnO/PFN-Br/active layer/PFN-Br/Ag and hole-only devices with the structure of ITO/MoO_3/active layer/ MoO_3/Ag are used to conduct SCLC measurements. The mobilities were determined by fitting the dark-field current density voltage curves using the Mott-Gurney relationship, which is described in the following equation,

$$J(V) = \frac{9}{8}\varepsilon_0 \varepsilon_r \mu_0 \frac{V^2}{L^3} \qquad (2)$$

where J is the current density, ε_0 is the permittivity of free space, ε_r is the relative permittivity of the material, μ_0 is the zero-field mobility, V is the effective voltage and L is the thickness of the active layer. From the plot of $J^{1/2}$ versus V, the hole and electron mobilities can be deduced.

The calculation processes of E_{loss}. The equations used for E_{loss} calculation are described as follow:

1. Radiative recombination above the bandgap (ΔE_1)

$$\Delta E_1 = E_g - qV_{OC}^{SQ} \tag{3}$$

$$V_{OC}^{SQ} = \frac{kT}{q}\ln\left(\frac{J_{SC}}{J_0^{SQ}} + 1\right) = \frac{kT}{q}\ln\left(\frac{q\int_0^{\infty} EQE_{PV}(E)\emptyset_{AM\,1.5}(E)dE}{q\int_{E_g}^{\infty}\emptyset_{BB}(E)dE} + 1\right) \tag{4}$$

$$\emptyset_{BB}(E) = \frac{2\pi}{h^3c^2}E^2\mathrm{e}^{-\frac{E}{KT}} \tag{5}$$

2. Radiative recombination below the bandgap (ΔE_2)

$$\Delta E_2 = E_{loss,rad} = qV_{OC}^{SQ} - qV_{OC}^{rad} \tag{6}$$

$$V_{OC}^{rad} = \frac{kT}{q}\ln\left(\frac{J_{SC}}{J_0^{rad}} + 1\right) = \frac{kT}{q}\ln\left(\frac{q\int_0^{\infty} EQE_{PV}(E)\emptyset_{AM\,1.5}(E)dE}{q\int_{E_0}^{\infty}\emptyset_{BB}(E)dE} + 1\right) \tag{7}$$

3. Non-radiative recombination loss (ΔE_3)

$$\Delta E_3 = E_{loss,non-rad} = -\frac{kT}{q}\ln EQE_{EL} \tag{8}$$

$$\Delta E_3^{cal} = E_g - qV_{OC} - \Delta E_1 - \Delta E_2 \tag{9}$$

where $\mathrm{E_g}$, $\mathrm{V_{OC}^{SQ}}$, k, T, q, $\emptyset_{\mathrm{BB}}$, and $\mathrm{V_{OC}^{rad}}$ are energy bandgap, Shockley-Queisser (SQ) open-circuit voltage limit, the Boltzmann constant, the temperature, the elementary charge, the black body spectrum and radiative recombination open-circuit voltage limit, respectively[1].

Supplementary Figures

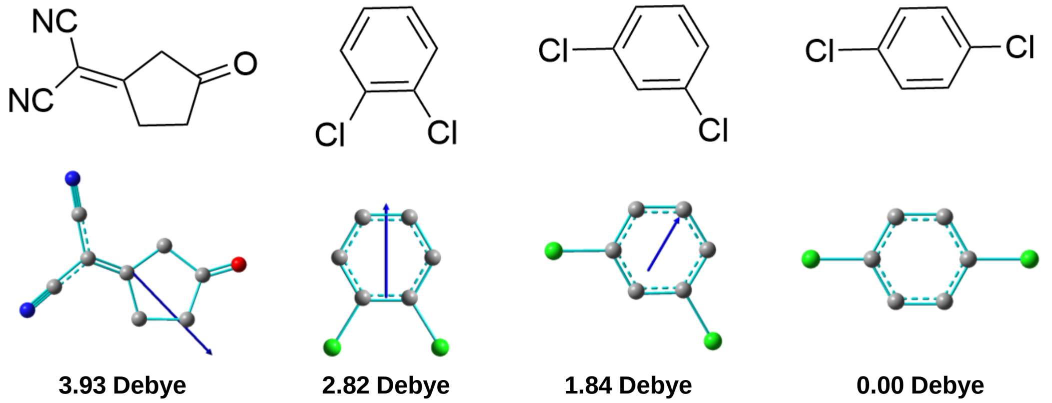

Supplementary Fig. 1 Chemical structures and dipole moments of 2-(4-oxocyclopent-2-en-1-ylidene) malononitrile and dichlorobenzene units.

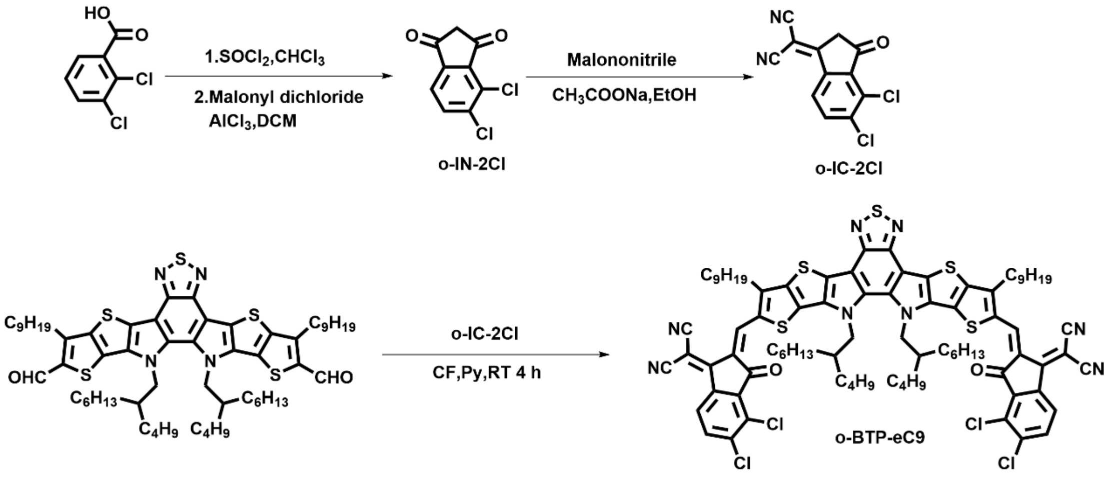

Supplementary Fig. 2 Synthetic methods of o-IN-2Cl, o-IC-2Cl, and o-BTP-eC9.

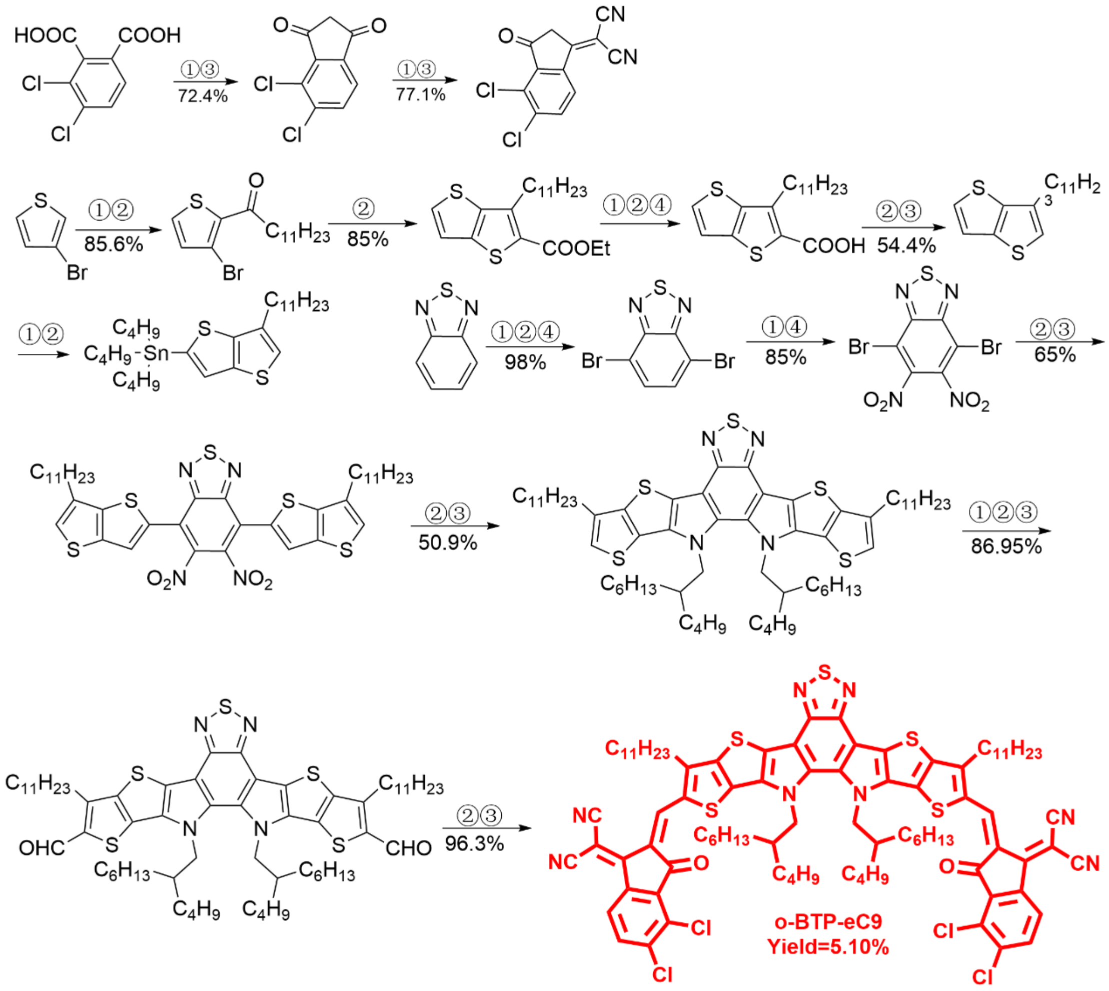

Supplementary Fig. 3 Synthetic route of o-BTP-eC9.

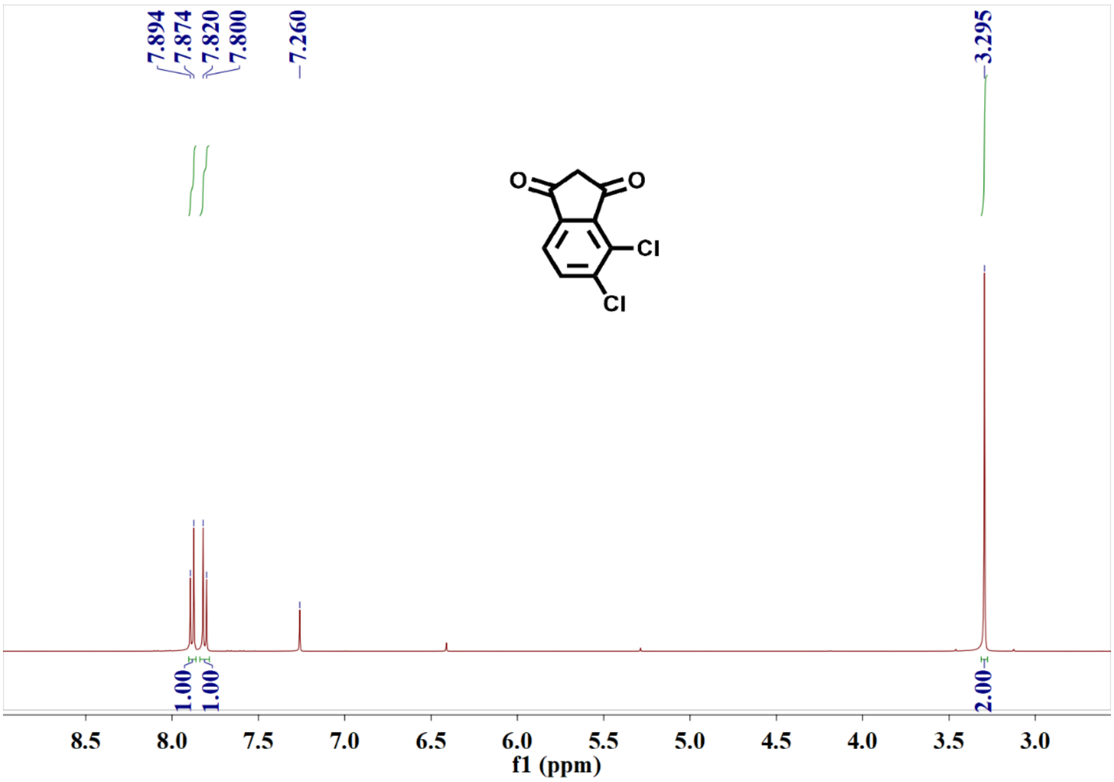

Supplementary Fig. 4 ^1H NMR spectrum of o-IN-2Cl.

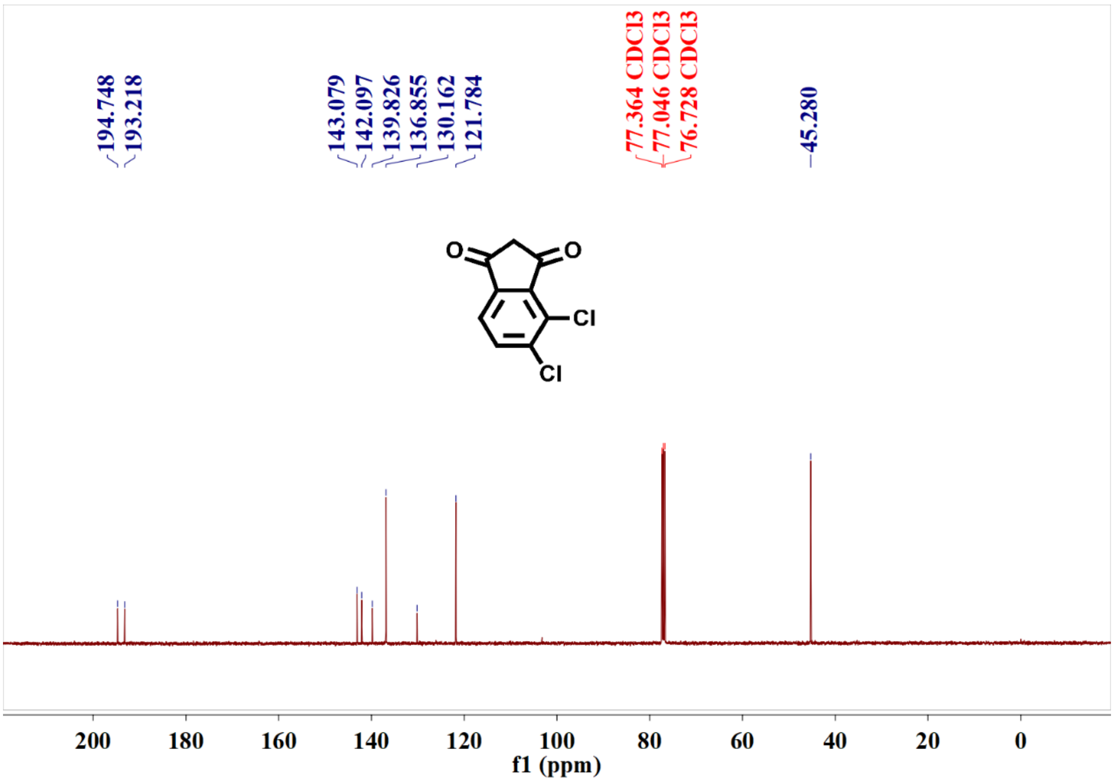

Supplementary Fig. 5 ^{13}C NMR spectrum of o-IN-2Cl.

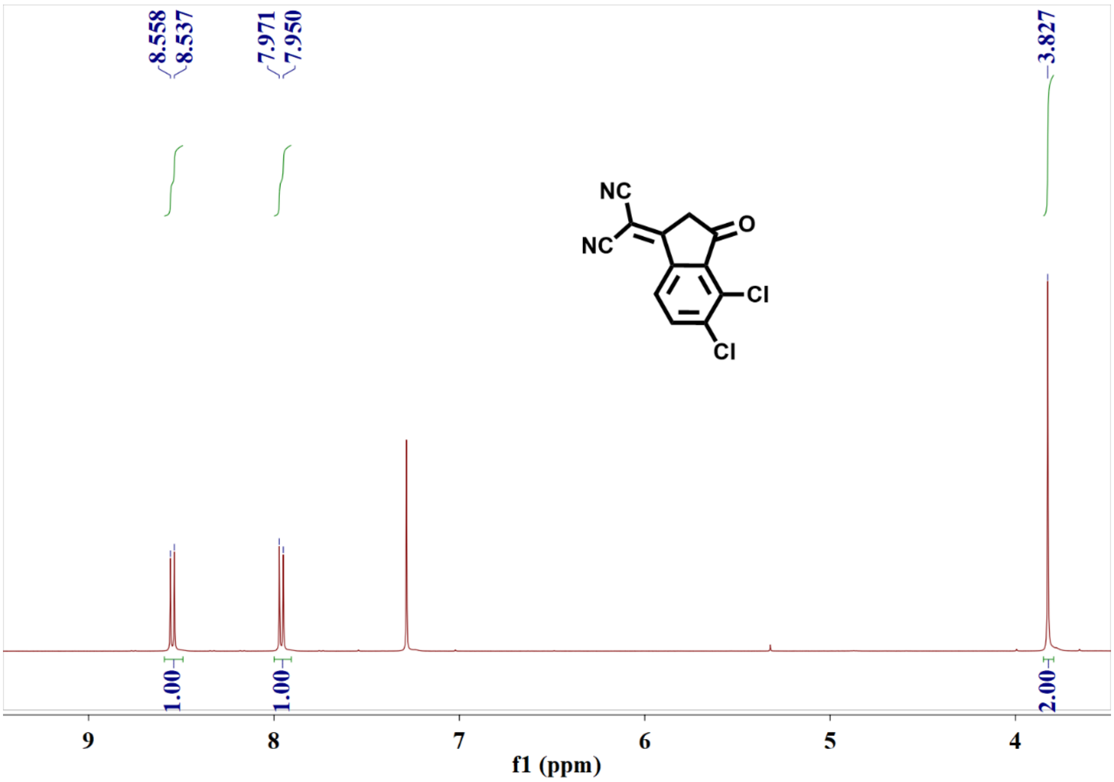

Supplementary Fig. 6 ^{1}H NMR spectrum of o-IC-2Cl.

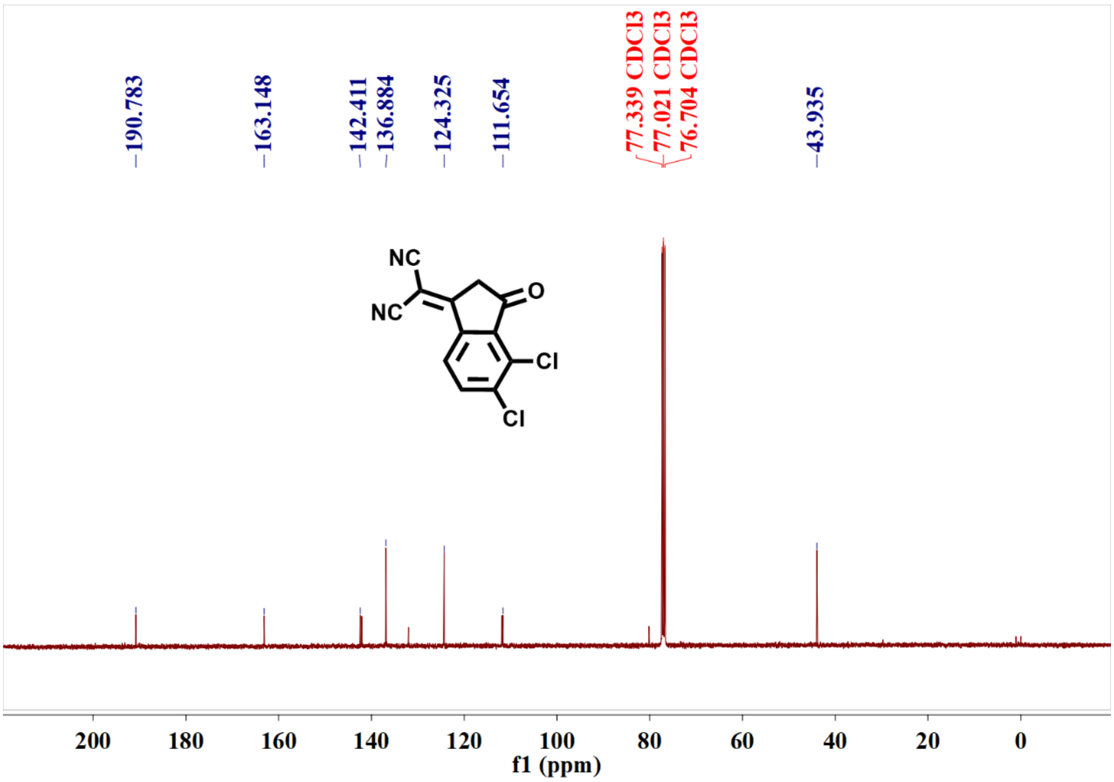

Supplementary Fig. 7 ^{13}C NMR spectrum of o-IC-2Cl.

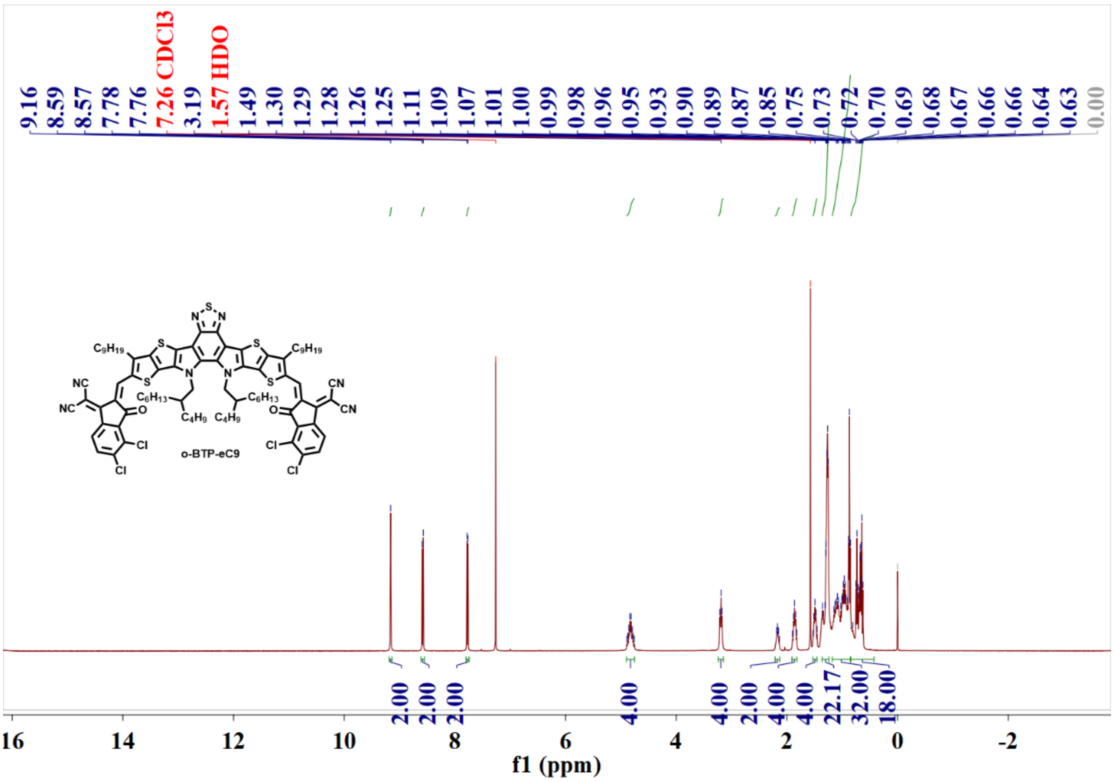

Supplementary Fig. 8 ^{1}H NMR spectrum of o-BTP-eC9.

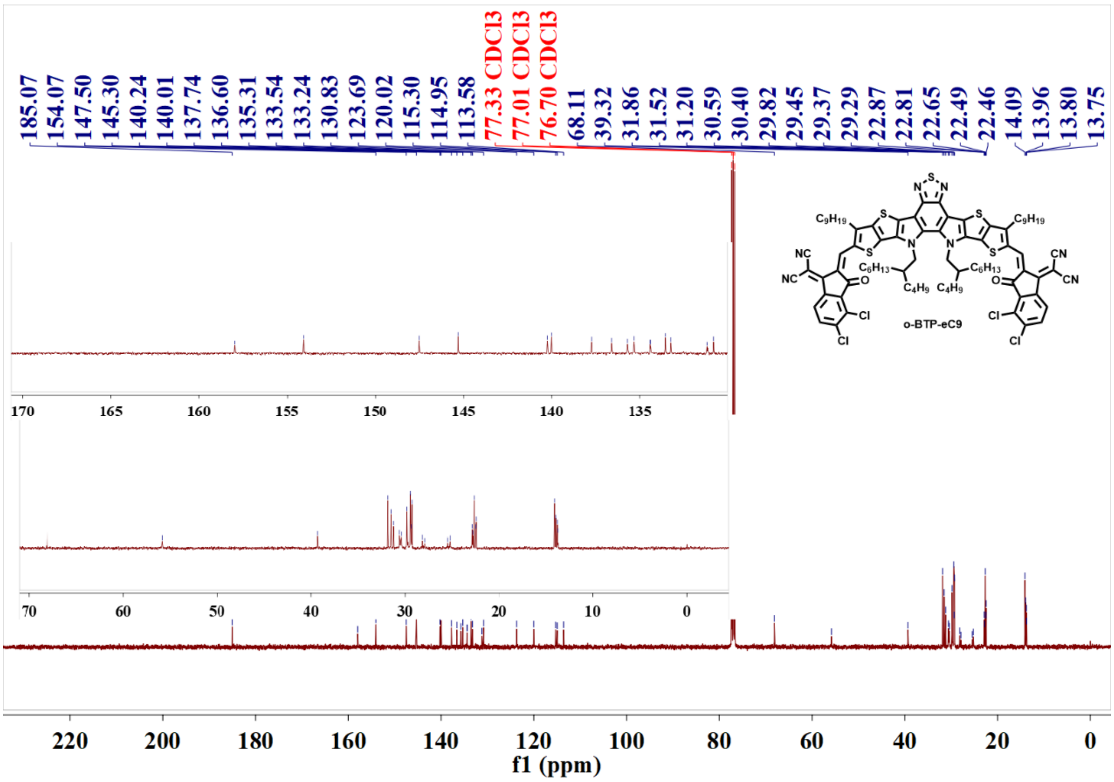

Supplementary Fig. 9 ^{13}C NMR spectrum of o-BTP-eC9.

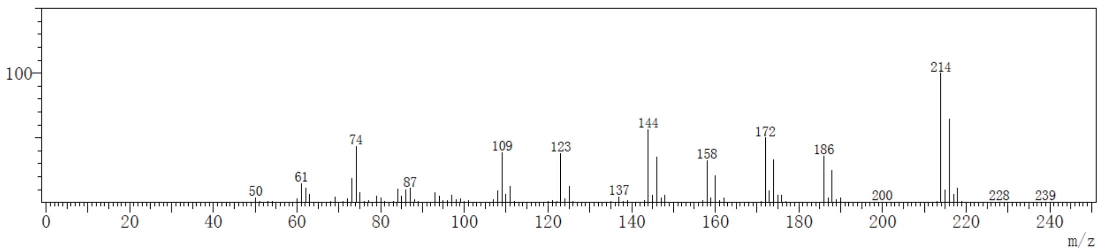

Supplementary Fig. 10 Gas chromatography-mass spectrometer (GCMS) Spectrum of o-IN-2Cl.

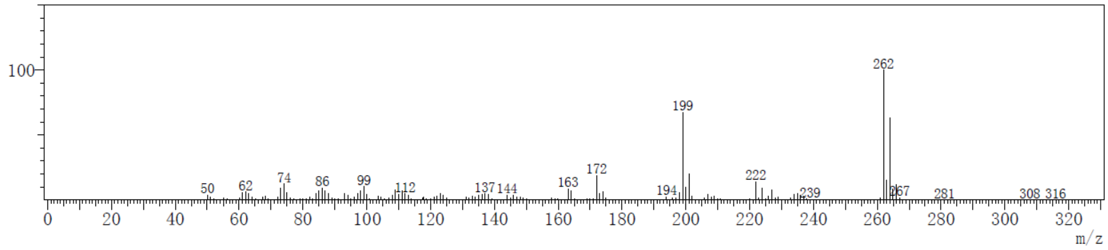

Supplementary Fig. 11 Gas chromatography-mass spectrometer (GCMS) spectrum of o-IC-2Cl.

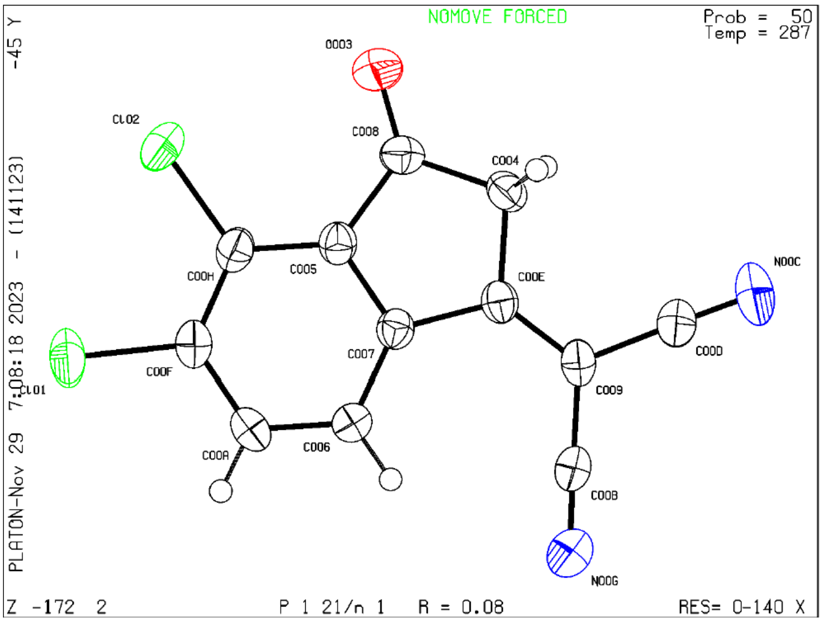

Supplementary Fig. 12 The chemical structure of o-IC-2Cl from single crystal analysis (CCDC number: 2312645). Note for the related B-level alert: Numerous crystals from many syntheses were tried, but all the crystals are of low quality. Despite long exposure times, the crystal did not exhibit observable intensity to a resolution higher than 0.90 angstrom.

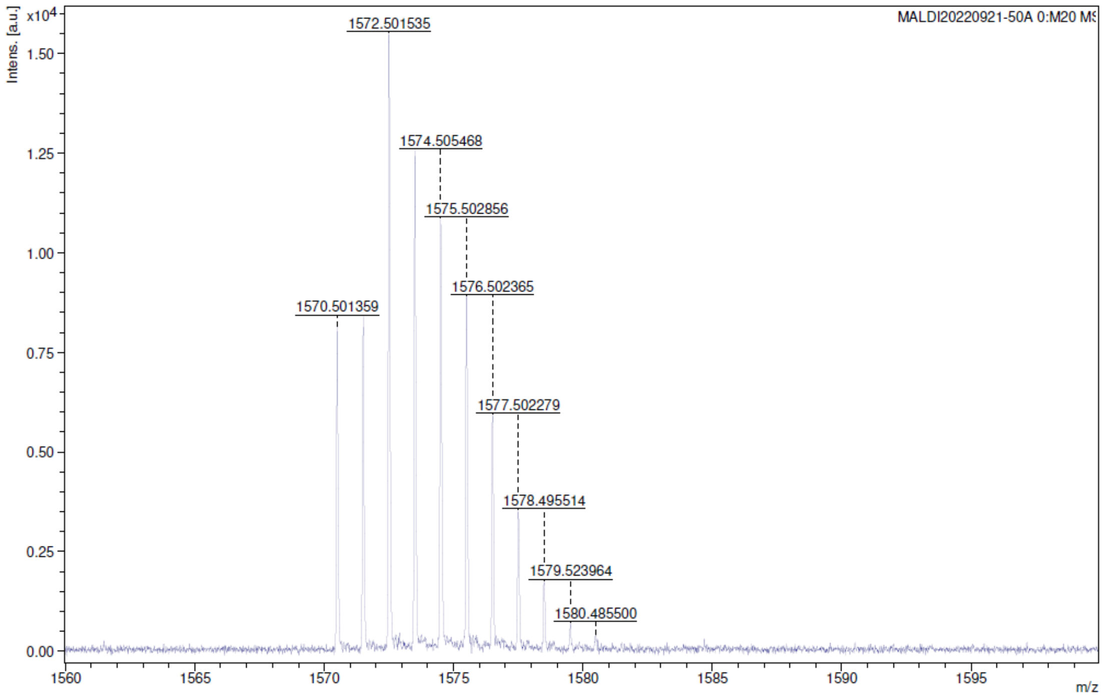

Supplementary Fig. 13 Matrix-assisted laser desorption/ionization (MALDI) time-of-flight (TOF) mass spectrometry (MS) of the o-BTP-eC9.

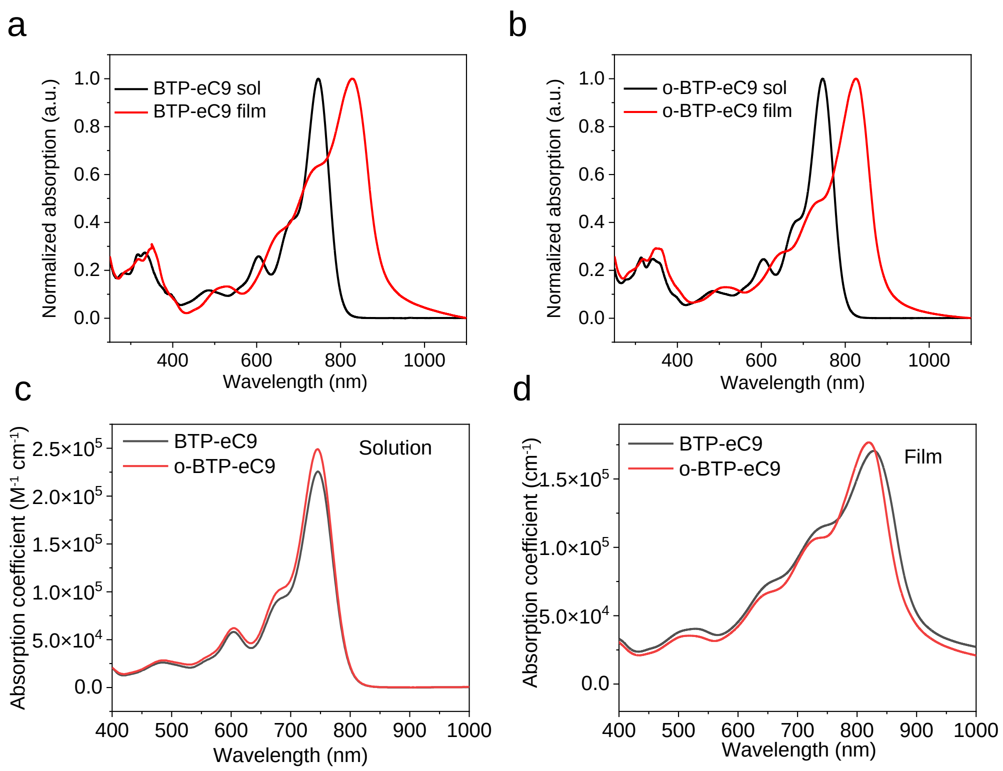

Supplementary Fig. 14 Normalized absorption spectra of BTP-eC9 (**a**) and o-BTP-eC9 (**b**) in solution (chloroform) state and film state. And absorption coefficient spectra of BTP-eC9 and o-BTP-eC9 in solution state (chloroform, **c**) and film state (**d**).

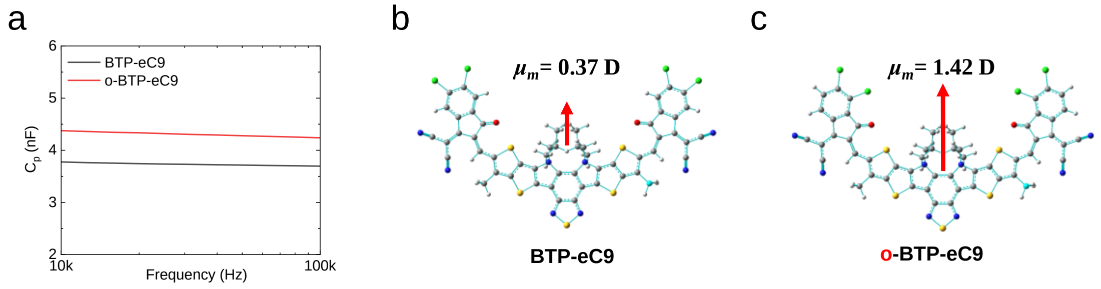

Supplementary Fig. 15 a, Flat C_p response of BTP-eC9 and o-BTP-eC9 films at different frequencies. Quantum chemistry calculation of the dipole moments of BTP-eC9 (**b**) and o-BTP-eC9 (**c**).

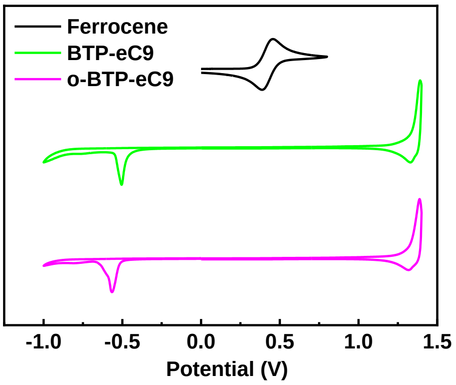

Supplementary Fig. 16 CV curves of ferrocene, BTP-eC9 and o-BTP-eC9.

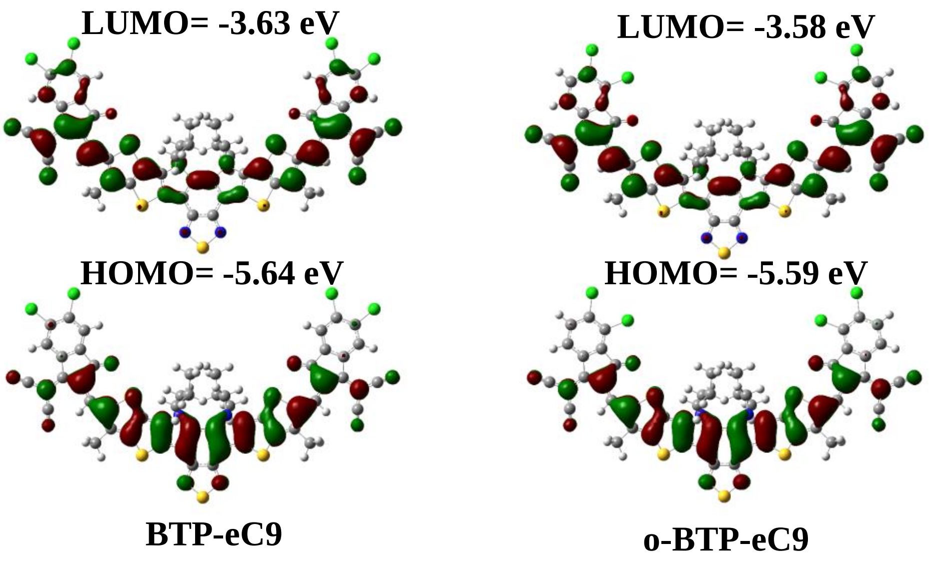

Supplementary Fig. 17 The molecular backbone conformations and the HOMO and LUMO electron distributions of BTP-eC9 and o-BTP-eC9.

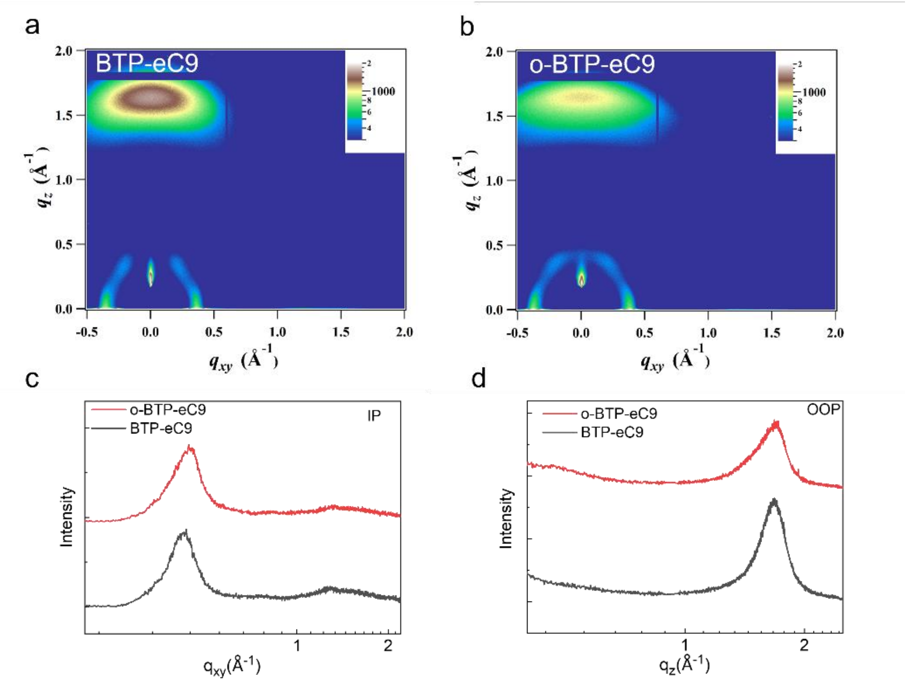

Supplementary Fig. 18 2D GIWAXS diffraction patterns of BTP-eC9 (**a**) and o-BTP-eC9 (**b**). And the corresponding 1D GIWAXS intensity profiles along in-plane (IP) direction (**c**) and out-of-plane (OOP) direction (**d**).

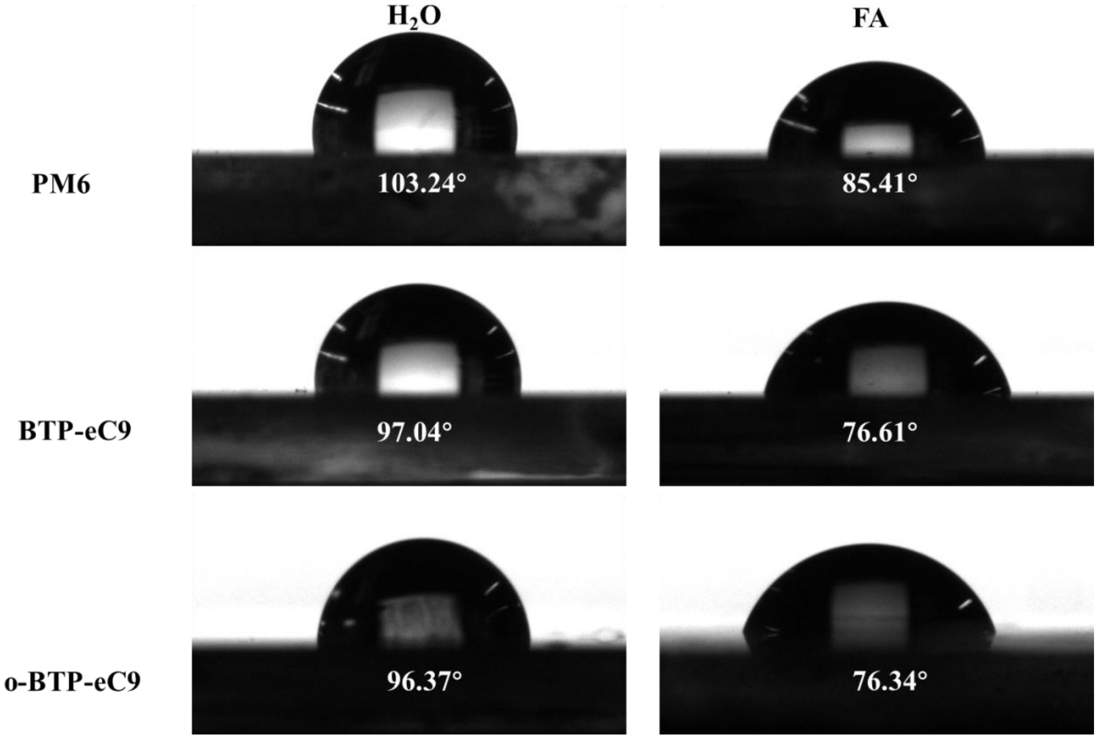

Supplementary Fig. 19 The contact angle images of water and glycerol on PM6, BTP-eC9 and o-BTP-eC9 films.

光焱科技光電校正實驗室
Enli Tech. Optoelectronic Calibration Lab.

Report No. : PVT220901

測試報告

Report of Test

儀器名稱 Device Name	Solar Cell
廠牌型號 Model No.	Organic Photovoltaic Solar Cells
儀器序號 Serial No.	C383
測試日期 Test Date	2022 / 06 / 24
送測單位 Applicant	Department of Electronic and Information Engineering, Research Institute for Smart Energy (RISE), The Hong Kong Polytechnic University
送測單位地址 Applicant Address	The Hong Kong Polytechnic University, Hung Hom, Kowloon, Hong Kong

上項儀器經本實驗室量測，結果如內文。本報告含封面及內文共 6 頁，分離使用無效。

The test device is measured by the laboratory and the results are given in the content. The report consists of 6 pages including the cover and is invalid if separated.

Enli Technology Co. Ltd.

報告簽署人/Approved by：

Hong Hom Wu

報告簽署暨發行日期/Date： 2022. 9. 30

實驗室地址(Laboratory Address)：

高雄市路竹區路科五路 96 號 1 樓 A 區 (A area, 1F., No.96, Luke 5th Rd., Kaohsiung, Taiwan, R.O.C)

TEL : +886-7-6955669 FAX : +886-7-6955785

測試報告使用說明 / Report Instruction

1. 光焱科技光電校正實驗室執行測試所產生之測試結果詳列於本報告內。

   本報告之測試結果僅對報告內提及之送測件有效。

   The tested results of the device are given in the report.
   The tested results of this report are responsible to the tested device.

2. 除特別聲明外，報告內數值係在本實驗室環境下執行測試所得的結果。

   爾後使用該送測件時，其準確度與精密度將因使用時之環境狀況與使用頻率等因素而有所不同。

   Numeric data in this report are the tested results in the ambient condition of the laboratory except special statements.
   Precision and accuracy of the test device will depend on the ambient condition and usage frequency of it afterward.

3. 本報告之結果業經本實驗室之主管審核確認。

   The calibration report should be approved by head of the calibration laboratory.

4. 本報告未得到本實驗室書面同意，不得任意摘錄複製使用，但全文複製除外。

   The calibration report should not be reproduced expect in full.

(以下空白/Blank)

Enli Technology Co. Ltd.

TEL: +886-7-6955669 FAX: +886-7-6955785

測試環境條件 Environment condition

模擬器種類 Simulator type	Steady-State Class A
照度 Irradiance	1000 W/m^2
待測件溫度 DUT Temperature	24.9 ℃
環境溫度 Environmental Temperature	26.0 ℃

測試使用之標準件 Standard for Calibration

儀器名稱 Device Name	Reference Cell		
廠牌型號 Model No.	SRC2020		
儀器序號 Serial No.	SRC-00170		
校正機構 Cal. Laboratory	NREL		
報告編號 Report No.	2037		
追溯日期 Cal. Date	2021 / 05 / 06		

標準件之溯源 Standard Traceability

儀器名稱 Device Name	Primary Reference cell		
廠牌型號 Model No.			
儀器序號 Serial No.	S09		
校正機構 Cal. Laboratory	NIST		
報告編號 Report No.			
追溯日期 Cal. Date	2020 / 04 / 07		
有效日期 Due Date			

Enli Technology Co. Ltd.

測試結果 Test Results

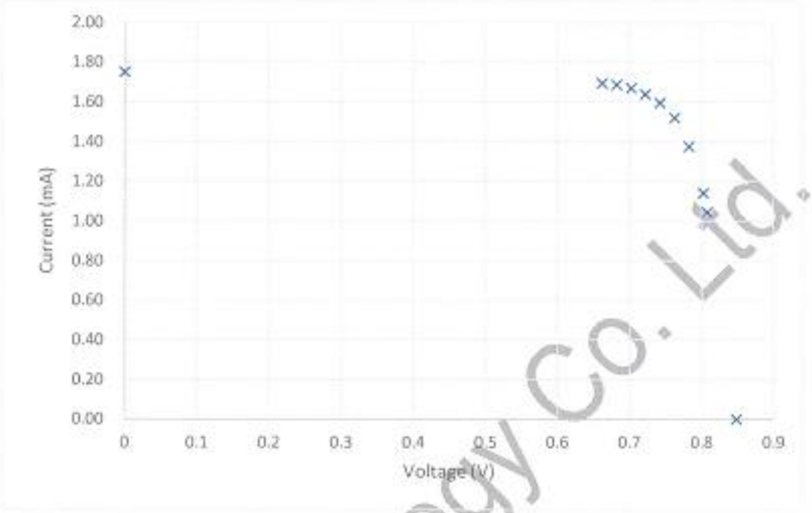

V_{OC}	=	847.86	mV	±	3.39	mV
I_{SC}	=	1749.70	μA	±	24.84	μA
P_{MPP}	=	1184.77	μW	±	17.41	μW
V_{MPP}	=	742.00	mV	±	2.97	mV
I_{MPP}	=	1596.73	μA	±	22.67	μA
FF	=	79.86		±	0.24	%
Efficiency	=	19.48		±	0.30	%
Aperture area	=	6.08	mm^2			

Asymptotic Pmax Scan

Scan duration time: 15.5 s.

Enli Technology Co. Ltd.

TEL : +886-7-6955669 FAX : +886-7-6955785

測試說明 Description of the test object

1 測試日期 Test Date：
   1.1 測試件接收日期 Date of Receipt：2022 / 06 / 22
   1.2 測試件校正日期 Date of Test：2022 / 06 / 24

2 測試地點 Test Site：
   2.1 光焱科技光電校正實驗室 Enli Tech. Optoelectronic Calibration Lab.

3 測試方法 Test Method：
   3.1 待測件的測試是依據本實驗室「太陽能電池最大功率測試作業指導書」[1]執行，於「IEC 60904-1 Photovoltaic devices - Part 1: Measurement of photovoltaic current-voltage characteristics」所定義之標準測試條件(STC)下進行測試，使用「IEC 60904-9 Photovoltaic devices - Part 9: Classification of solar simulator characteristics」所定義之 3A 等級穩態太陽光模擬器進行輻照，並根據「IEC 60904-7 Photovoltaic devices - Part 7: Computation of the spectral mismatch correction for measurements of photovoltaic devices」來計算光譜失配係數。太陽光譜模擬器的光譜使用光譜儀測量，並依據「IEC 60904-8 Photovoltaic devices - Part 8: Measurement of spectral responsivity of a photovoltaic (PV) device」使用單光儀測量待測件的光譜響應（或量子效率）。

   The testing of the DUT was performed at Standard Testing Conditions (STC) in accordance with IEC 60904-1 Photovoltaic devices - Part 1: Measurement of photovoltaic current-voltage characteristics and Test standard operation procedure of maximum power measurement of solar cells [1] under the irradiation with a steady-state class AAA solar simulator according to IEC 60904-9 Photovoltaic devices - Part 9: Classification of solar simulator characteristics. The spectral mismatch is calculated according to IEC 60904-7 Photovoltaic devices - Part 7: Computation of the spectral mismatch correction for measurements of photovoltaic devices. The spectrum of the solar simulator is measured with a spectroradiometer. The spectral responsivity (or quantum efficiency) of the device under test is measured with a grating monochromatic according to IEC 60904-8 Photovoltaic devices - Part 8: Measurement of spectral responsivity of a photovoltaic (PV) device.

4 相對擴充不確定度 Relative Expanded Uncertainty:
   4.1 相對擴充不確定度係依據本實驗室之「最大功率測試量測不確定度評估報告」[2]進行評估。

   Relative expanded uncertainty is estimated based on *Estimated Uncertainty Report of maximum power measurement of solar cells [2].*

Enli Technology Co., Ltd.

測試說明 Description of the test object

1 測試日期 Test Date：
 1.1 測試件接收日期 Date of Receipt：2022 / 06 / 22
 1.2 測試件校正日期 Date of Test：2022 / 06 / 24

2 測試地點 Test Site：
 2.1 光焱科技光電校正實驗室 Enli Tech. Optoelectronic Calibration Lab.

3 測試方法 Test Method：
 3.1 待測件的測試是依據本實驗室「太陽能電池最大功率測試作業指導書」[1]執行，於「IEC 60904-1 Photovoltaic devices - Part 1: Measurement of photovoltaic current-voltage characteristics」所定義之標準測試條件(STC)下進行測試，使用「IEC 60904-9 Photovoltaic devices - Part 9: Classification of solar simulator characteristics」所定義之 3A 等級穩態太陽光模擬器進行輻照，並根據「IEC 60904-7 Photovoltaic devices - Part 7: Computation of the spectral mismatch correction for measurements of photovoltaic devices」來計算光譜失配係數。太陽光譜模擬器的光譜使用光譜儀測量，並依據「IEC 60904-8 Photovoltaic devices - Part 8: Measurement of spectral responsivity of a photovoltaic (PV) device」使用單光儀測量待測件的光譜響應（或量子效率）。

 The testing of the DUT was performed at Standard Testing Conditions (STC) in accordance with IEC 60904-1 Photovoltaic devices - Part 1: Measurement of photovoltaic current-voltage characteristics and Test standard operation procedure of maximum power measurement of solar cells [1] under the irradiation with a steady-state class AAA solar simulator according to IEC 60904-9 Photovoltaic devices - Part 9: Classification of solar simulator characteristics. The spectral mismatch is calculated according to IEC 60904-7 Photovoltaic devices - Part 7: Computation of the spectral mismatch correction for measurements of photovoltaic devices. The spectrum of the solar simulator is measured with a spectroradiometer. The spectral responsivity (or quantum efficiency) of the device under test is measured with a grating monochromatic according to IEC 60904-8 Photovoltaic devices - Part 8: Measurement of spectral responsivity of a photovoltaic (PV) device.

Enli Technology Co., Ltd.

4 相對擴充不確定度 Relative Expanded Uncertainty:
 4.1 相對擴充不確定度係依據本實驗室之「最大功率測試量測不確定度評估報告」[2]進行評估。

 Relative expanded uncertainty is estimated based on *Estimated Uncertainty Report of maximum power measurement of solar cells [2].*

TEL : +886-7-6955669 FAX : +886-7-6955785

4.2 相對擴充不確定度係相對組合標準不確定度與涵蓋因子(*k*)之乘積，*k*=2 相對應約 95 % 之信賴水準。

The relative expanded uncertainty resulting of the relative combined standard uncertainty multiplied with a coverage factor *k*=2 is specified. It corresponds to about a level of confidence of approximately 95 %.

參考文件 Reference Literature

1. LAB-3-7.2.2(1) 太陽能電池最大功率測試作業指導書
   LAB-3-7.2.2(1), *Test standard operation procedure of maximum power measurement of solar cells*, Enli Technology Co., Ltd.
2. LAB-3-7.6.2(1) 最大功率測試量測不確定度評估報告
   LAB-3-7.6.2(1), *Estimated Uncertainty Report of maximum power measurement of solar cells*, Enli Technology Co., Ltd.

（以下空白/Blank）

Enli Technology Co., Ltd.

TEL : +886-7-6955669 FAX : +886-7-6955785

Supplementary Fig. 20 Certified efficiency. The copy images of PCE certificate of PM6:BTP-eC9: o-BTP-eC9 based ternary OSC.

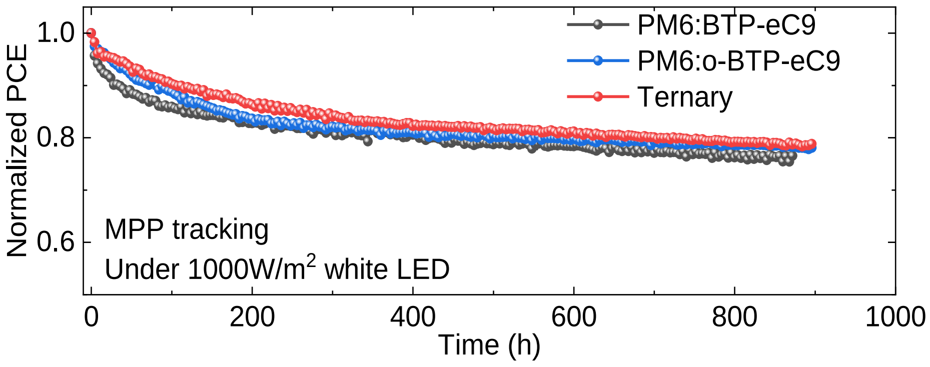

Supplementary Fig. 21 Light stability tests for binary and ternary OSCs, all devices were encapsulated and stored under continuous illumination equivalent to 1 sun in air.

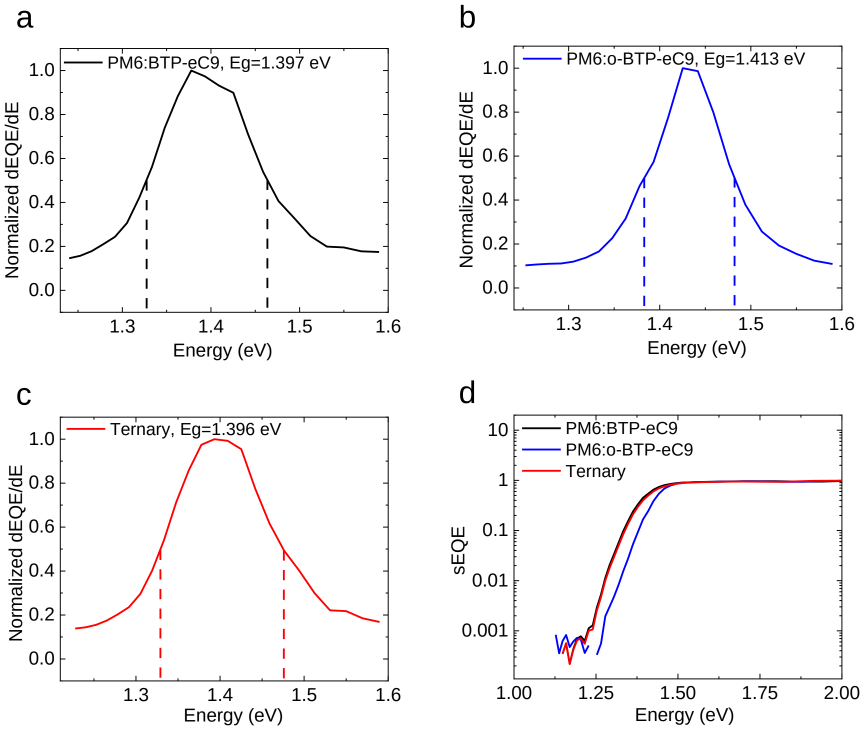

Supplementary Fig. 22 Determination of the optical bandgaps of the PM6:BTP-eC9 based device (**a**), the PM6:o-BTP-eC9 based device (**b**), and the ternary device(**c**). (**d**)Highly sensitive EQE (sEQE) curves of the binary and ternary devices.

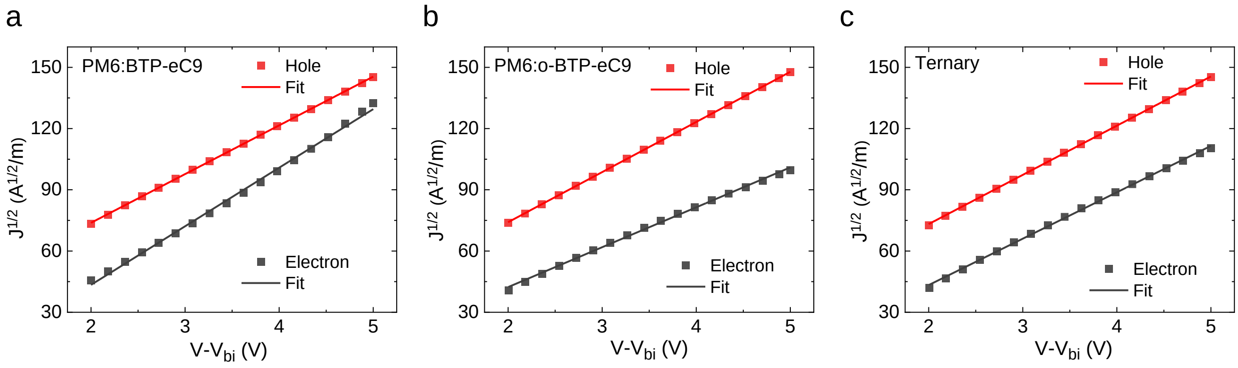

Supplementary Fig. 23 SCLC measurements. The dark $J^{1/2}$-V curves of the PM6:BTP-eC9 based device (**a**), the PM6:o-BTP-eC9 based device (**b**), and the ternary device(**c**).

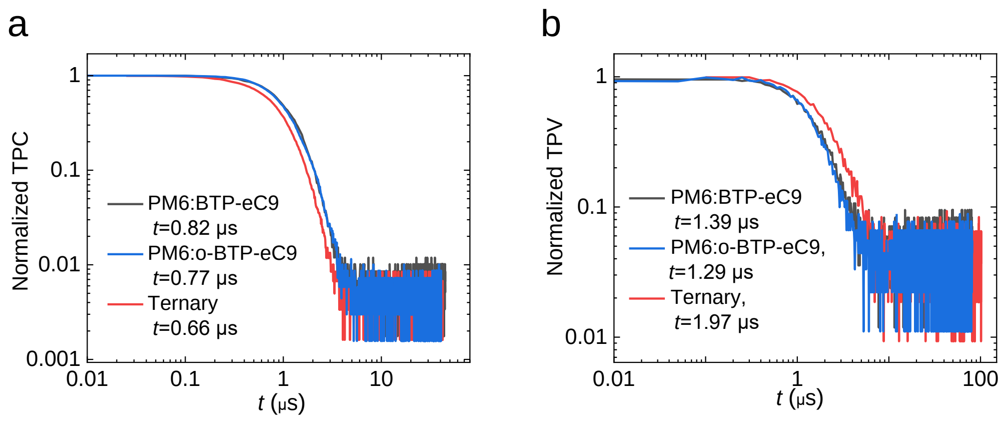

Supplementary Fig. 24 TPC (**a**) and TPV (**b**) measurements of the binary and ternary devices.

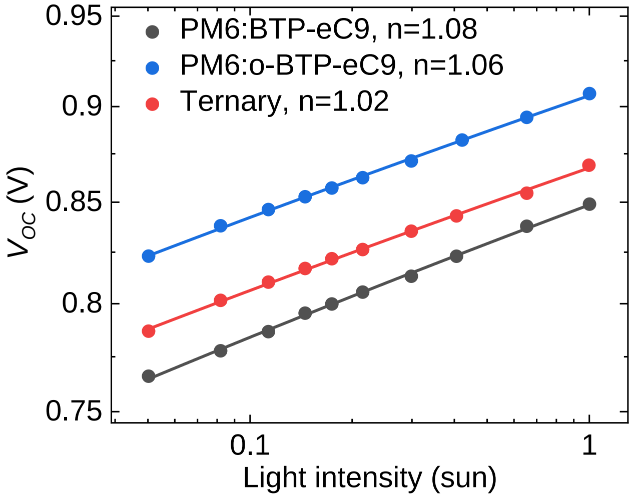

Supplementary Fig. 25 V_{OC} versus light intensity of the binary and ternary devices.

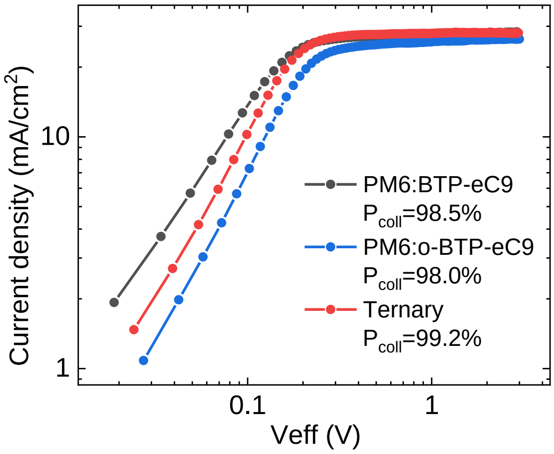

Supplementary Fig. 26 J_{ph}-V_{eff} curves of the binary and ternary devices.

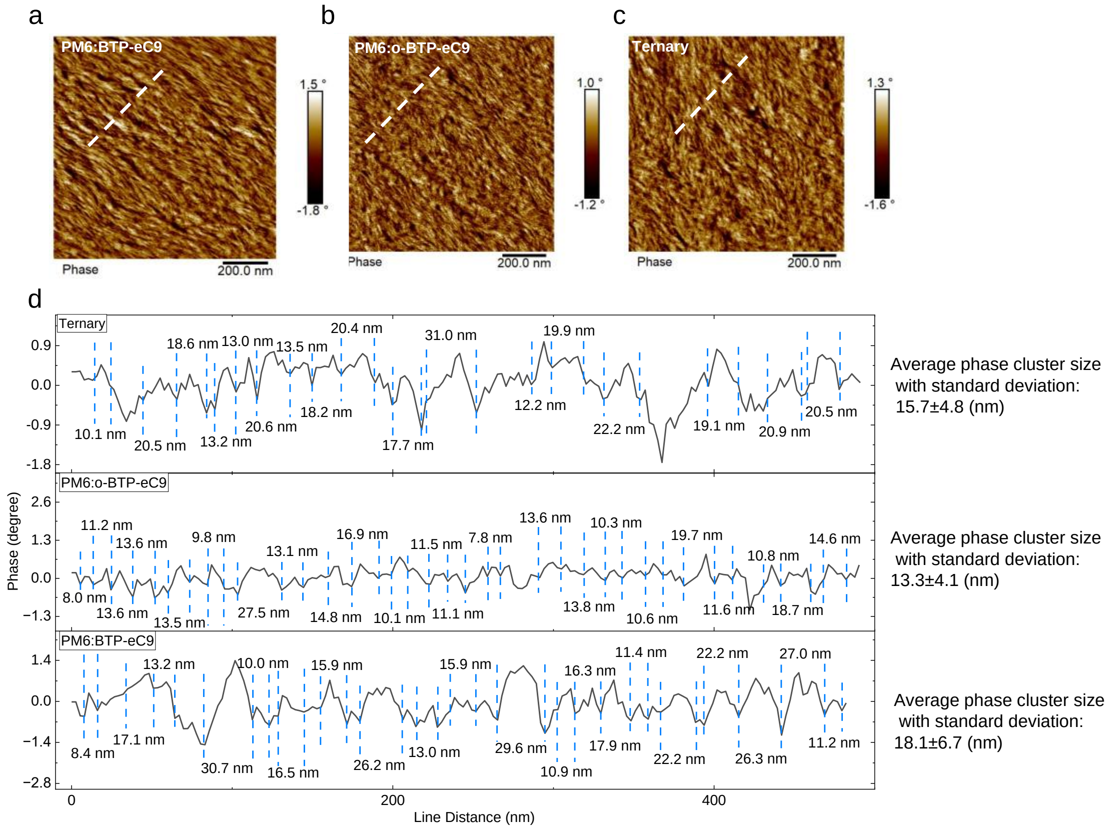

Supplementary Fig. 27 AFM phase images of PM6:BTP-eC9 (**a**), PM6:o-BTP-eC9 (**b**) and ternary (**c**) blend films. **d**, Line profile of phase images of PM6:BTP-eC9, PM6:o-BTP-eC9 and ternary blend films

Supplementary Tables

Supplementary Table 1. The surface energy values of PM6, BTP-eC9, and o-BTP-eC9.

Film	C_p (nF)	d (nm)	A (mm^2)	ε_r
BTP-eC9	3.8 (3.7±0.1)	65 (62±2)	11	2.5 (2.4±0.1)
o-BTP-eC9	4.3 (4.2±0.1)	67 (64±2)	11	3.0 (2.9±0.1)

Note: The average values with standard deviations in parentheses are calculated from 8 devices.

Supplementary Table 2. Detailed peak information from GIWAXS measurements of the neat films.

q_{xy}-lamellar peak	Position(Å^{-1})	*d*-spacing (Å)	FWHM (Å^{-1})	CCL (Å)
BTP-eC9	0.422	13.400	0.095	59.525
o-BTP-eC9	0.436	12.970	0.099	57.120
q_z-π-π stacking peak	Position (Å^{-1})	*d*-spacing (Å)	FWHM (Å^{-1})	CCL (Å)
BTP-eC9	1.680	3.366	0.311	18.183
o-BTP-eC9	1.684	3.358	0.369	15.325

Supplementary Table 3. The surface energy values of PM6, BTP-eC9, and o-BTP-eC9.

	Contact angle-water	Contact angle-glycerol	Surface energy (mN m^{-1})
PM6	103.5^{o}	83.8^{o}	20.71
BTP-eC9	97.4^{o}	76.5^{o}	24.94
o-BTP-eC9	96.1^{o}	75.2^{o}	25.57

Supplementary Table 4. Photovoltaic parameters of the ternary OSCs versus the ratio of o-BTP-eC9. Here PM6 is denoted as D, BTP-eC9 is denoted as A_1 and o-BTP-eC9 is denoted as A_2.

D: A_1: A_2	V_{OC} (V)	J_{SC} (mA cm^{-2})	FF (%)	PCE[a] (%)
1: 1.2: 0	0.843	28.27	79.19	18.87 (18.69±0.07)
1: 1.1: 0.1	0.854	28.36	79.73	19.31 (19.12±0.10)
1: 1.05: 0.15	0.860	28.75	80.41	19.88 (19.63±0.12)
1: 1.0: 0.2	0.865	28.24	79.86	19.51 (19.33±0.09)
1: 0.9: 0.3	0.876	27.76	79.28	19.26 (19.02±0.11)
1: 0: 1.2	0.901	26.33	78.83	18.71 (18.56±0.07)

[a]The average PCEs with standard deviation calculated from 20 devices. All devices were tested with a metal mask applied.

Supplementary Table 5. Charge mobilities measured by SCLC method for the binary and ternary devices.

Blend	μ_e ($cm^2V^{-1}s^{-1}$)	μ_h ($cm^2V^{-1}s^{-1}$)	μ_e/μ_h
PM6: BTP-eC9	5.1×10^{-4}	4.0×10^{-4}	1.28
PM6:o-BTP-eC9	3.0×10^{-4}	3.7×10^{-4}	0.81
Ternary	4.0×10^{-4}	3.8×10^{-4}	1.05

Supplementary Table 6. Detailed peak information from GIWAXS measurements of the binary and ternary blend films.

q_{xy}-lamellar peak	Position(Å^{-1})	*d*-spacing (Å)	FWHM (Å^{-1})	CCL (Å)
PM6: BTP-eC9	0.336	18.700	0.047	120.316
PM6: o-BTP-eC9	0.332	18.925	0.053	106.696
Ternary	0.336	18.700	0.046	122.932
PM6: BTP-eC9	0.404	15.552	0.115	49.173
PM6: o-BTP-eC9	0.421	14.924	0.140	40.392
Ternary	0.405	15.514	0.113	50.043
**q_z-π-π stacking peak**	**Position (Å^{-1})**	***d*-spacing (Å)**	**FWHM (Å^{-1})**	**CCL (Å)**
PM6: BTP-eC9	1.683	3.733	0.214	26.425
PM6: o-BTP-eC9	1.684	3.731	0.251	22.529
Ternary	1.678	3.744	0.229	24.694

Supplementary Reference

1 Liu, J. *et al.* Fast charge separation in a non-fullerene organic solar cell with a small driving force. *Nat. Energy* **1**, 16089 (2016).
