# Supplementary material for: Rational molecular and device design enables organic solar cells approaching 20% efficiency
Source: Nat Commun. 2024 Feb 28;15:1830. doi: 10.1038/s41467-024-46022-3 (PMC10902355; doi:10.1038/s41467-024-46022-3)
Supplement: Supplementary file 3 — Description of Additional Supplementary Files [file 41467_2024_46022_MOESM3_ESM.pdf]

File name: Supplementary Data 1
Description: The structure factors and structural output of the o-IC-2Cl single crystal checked by using CheckCIF routine.

File name: Supplementary Data 2
Description: The CIF file of the o-IC-2Cl single crystal.

File name: Supplementary Data 3
Description: The coordinates of the optimized computational models for BTP-eC9 and o-BTP-eC9.
