# Supplementary material for: Rational molecular and device design enables organic solar cells approaching 20% efficiency
Source: Nat Commun. 2024 Feb 28;15:1830. doi: 10.1038/s41467-024-46022-3 (PMC10902355; doi:10.1038/s41467-024-46022-3)

checkCIF/PLATON report

Structure factors have been supplied for datablock(s) 2

THIS REPORT IS FOR GUIDANCE ONLY. IF USED AS PART OF A REVIEW PROCEDURE FOR PUBLICATION, IT SHOULD NOT REPLACE THE EXPERTISE OF AN EXPERIENCED CRYSTALLOGRAPHIC REFEREE.

No syntax errors found. CIF dictionary Interpreting this report

Datablock: 2

```
Bond precision:   C-C = 0.0040 A          Wavelength=1.54178

Cell:              a=9.797(2)     b=6.8991(14)        c=16.745(3)
                   alpha=90       beta=94.343(8)      gamma=90
Temperature:       287 K

                Calculated                  Reported
Volume          1128.6(4)                   1128.5(4)
Space group     P 21/n                      P 1 21/n 1
Hall group      -P 2yn                      -P 2yn
Moiety formula  C12 H4 Cl2 N2 O             C12 H4 Cl2 N2 O
Sum formula     C12 H4 Cl2 N2 O             C12 H4 Cl2 N2 O
Mr              263.07                      263.07
Dx,g cm-3       1.548                       1.548
Z               4                           4
Mu (mm-1)       5.037                       5.037
F000            528.0                       528.0
F000'           532.18
h,k,lmax                                    10,7,18
Nref                                        1618
Tmin,Tmax                                   0.402,0.752
Tmin'

Correction method= # Reported T Limits: Tmin=0.402 Tmax=0.752
AbsCorr = NONE

Data completeness=                 Theta(max)= 58.993

R(reflections)= 0.0818( 1542)                         wR2(reflections)=
                                                      0.2229( 1618)
S = 1.297                  Npar= 154
```

The following ALERTS were generated. Each ALERT has the format
test-name_ALERT_alert-type_alert-level.
Click on the hyperlinks for more details of the test.

Alert level B

```
THETM01_ALERT_3_B  The value of sine(theta_max)/wavelength is less than 0.575
            Calculated sin(theta_max)/wavelength =    0.5559
```

Alert level C

```
DIFMN02_ALERT_2_C  The minimum difference density is < -0.1*ZMAX*0.75
            _refine_diff_density_min given =     -1.461
            Test value =     -1.275
DIFMN03_ALERT_1_C  The minimum difference density is < -0.1*ZMAX*0.75
            The relevant atom site should be identified.
PLAT053_ALERT_1_C Minimum Crystal Dimension Missing (or Error) ...     Please Check
PLAT054_ALERT_1_C Medium  Crystal Dimension Missing (or Error) ...     Please Check
PLAT055_ALERT_1_C Maximum Crystal Dimension Missing (or Error) ...     Please Check
PLAT098_ALERT_2_C Large Reported Min.  (Negative) Residual Density      -1.46 eA-3
PLAT911_ALERT_3_C Missing FCF Refl Between Thmin & STh/L=    0.556          9 Report
               -1  1  1,   1  0  1,   2  1  2,  -1  0  3,  -4  0  4,   4  2  5,
               -2  7  7,   8  4  7,   9  1  9,
PLAT918_ALERT_3_C Reflection(s) with I(obs) much Smaller I(calc) .          8 Check
PLAT939_ALERT_3_C Large Value of Not (SHELXL) Weight Optimized S .      77.63 Check
```

Alert level G

```
PLAT003_ALERT_2_G Number of Uiso or Uij Restrained non-H Atoms ...          2 Report
PLAT072_ALERT_2_G SHELXL First  Parameter in WGHT  Unusually Large       0.16 Report
PLAT186_ALERT_4_G The CIF-Embedded .res File Contains ISOR Records          1 Report
PLAT720_ALERT_4_G Number of Unusual/Non-Standard Labels ..........         21 Note
              Cl01    Cl02    O003    C004    H00A    H00B    C005    C006
              H006    C007    C008    C009    C00A    H00C    C00B    N00C
              C00D    C00E    C00F    N00G    C00H
PLAT860_ALERT_3_G Number of Least-Squares Restraints .............         12 Note
PLAT909_ALERT_3_G Percentage of I>2sig(I) Data at Theta(Max) Still        92% Note
PLAT913_ALERT_3_G Missing # of Very Strong Reflections in FCF ....          1 Note
                2  1  2,
PLAT967_ALERT_5_G Note: Two-Theta Cutoff Value in Embedded .res ..      120.0 Degree
PLAT978_ALERT_2_G Number C-C Bonds with Positive Residual Density.          0 Info
```

0 **ALERT level A** = Most likely a serious problem - resolve or explain
1 **ALERT level B** = A potentially serious problem, consider carefully
9 **ALERT level C** = Check. Ensure it is not caused by an omission or oversight
9 **ALERT level G** = General information/check it is not something unexpected

4 ALERT type 1 CIF construction/syntax error, inconsistent or missing data
5 ALERT type 2 Indicator that the structure model may be wrong or deficient
7 ALERT type 3 Indicator that the structure quality may be low
2 ALERT type 4 Improvement, methodology, query or suggestion
1 ALERT type 5 Informative message, check

It is advisable to attempt to resolve as many as possible of the alerts in all categories. Often the minor alerts point to easily fixed oversights, errors and omissions in your CIF or refinement strategy, so attention to these fine details can be worthwhile. In order to resolve some of the more serious problems it may be necessary to carry out additional measurements or structure refinements. However, the purpose of your study may justify the reported deviations and the more serious of these should normally be commented upon in the discussion or experimental section of a paper or in the "special_details" fields of the CIF. checkCIF was carefully designed to identify outliers and unusual parameters, but every test has its limitations and alerts that are not important in a particular case may appear. Conversely, the absence of alerts does not guarantee there are no aspects of the results needing attention. It is up to the individual to critically assess their own results and, if necessary, seek expert advice.

Publication of your CIF in IUCr journals

A basic structural check has been run on your CIF. These basic checks will be run on all CIFs submitted for publication in IUCr journals (*Acta Crystallographica*, *Journal of Applied Crystallography*, *Journal of Synchrotron Radiation*); however, if you intend to submit to *Acta Crystallographica Section C* or *E* or *IUCrData*, you should make sure that full publication checks are run on the final version of your CIF prior to submission.

Publication of your CIF in other journals

Please refer to the *Notes for Authors* of the relevant journal for any special instructions relating to CIF submission.

PLATON version of 14/11/2023; check.def file version of 14/09/2023

Datablock 2 - ellipsoid plot

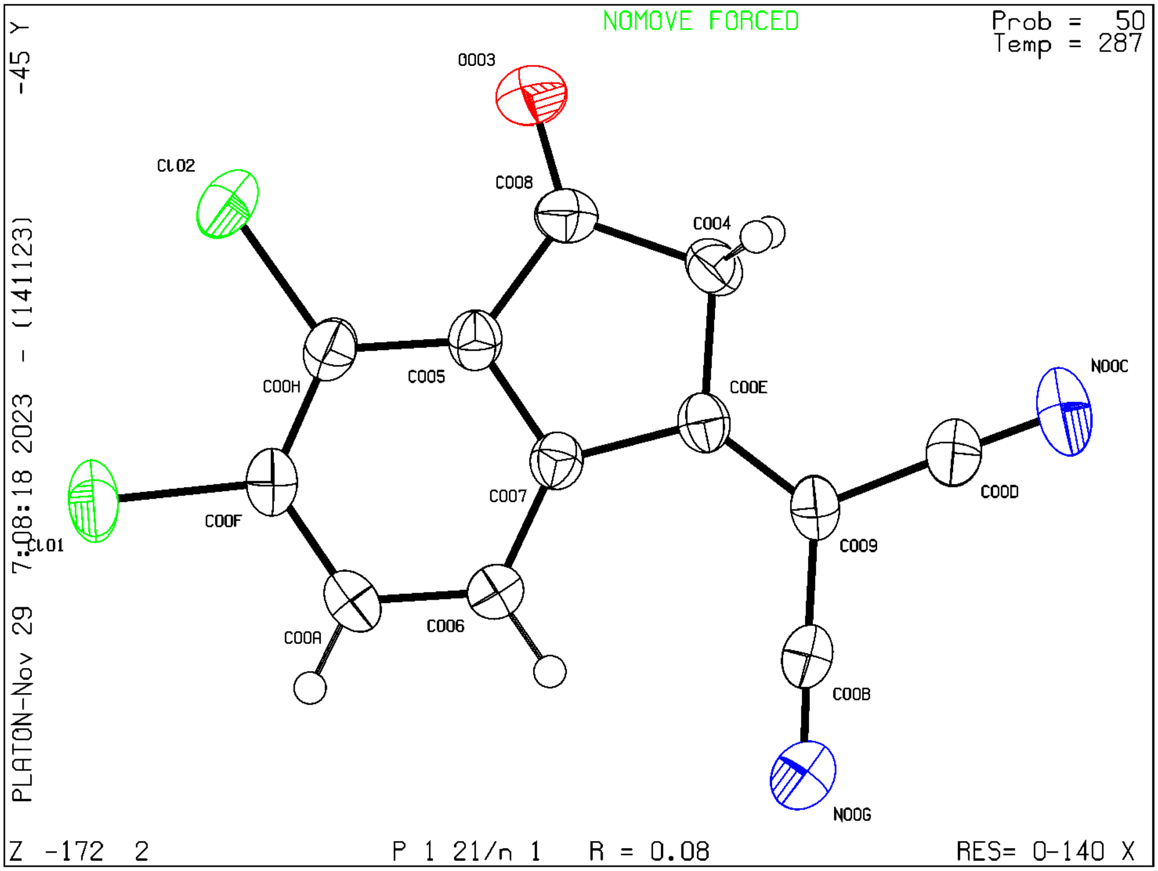

Supplement: Supplementary file 4 — Supplementary Data 1 [file 41467_2024_46022_MOESM4_ESM.pdf]
