# Supplementary material for: Rational molecular and device design enables organic solar cells approaching 20% efficiency
Source: Nat Commun. 2024 Feb 28;15:1830. doi: 10.1038/s41467-024-46022-3 (PMC10902355; doi:10.1038/s41467-024-46022-3)
Supplement: Supplementary file 7 — Reporting Summary [file 41467_2024_46022_MOESM7_ESM.pdf]

Corresponding author(s): Shirong Lu, Zeyun, Xiao and Gang Li

Solar Cells Reporting Summary

Nature Portfolio wishes to improve the reproducibility of the work that we publish. This form is intended for publication with all accepted papers reporting the characterization of photovoltaic devices and provides structure for consistency and transparency in reporting. Some list items might not apply to an individual manuscript, but all fields must be completed for clarity.

For further information on Nature Research policies, including our data availability policy, see Authors & Referees.

Experimental design

Please check the following details are reported in the manuscript, and provide a brief description or explanation where applicable.

1. Dimensions

Item	Yes/No	Description / Explanation
Area of the tested solar cells	☒ Yes ☐ No	Active area of the tested solar cells is 0.11cm2 / *Explain why this information is not reported/not relevant.*
Method used to determine the device area	☒ Yes ☐ No	The active area was determined by the crossed area of counter electrode and ITO stripe. / *Explain why this information is not reported/not relevant.*

2. Current-voltage characterization

Item	Yes/No	Description / Explanation
Current density-voltage (J-V) plots in both forward and backward direction	☐ Yes ☒ No	Generally, organic photovoltaic devices do not have hysteresis problem. And we only scan the device in forward direction.
Voltage scan conditions	☒ Yes ☐ No	Section "Device fabrication and testing" / *Explain why this information is not reported/not relevant.*
Test environment	☒ Yes ☐ No	In glove box at room temperature. / *Explain why this information is not reported/not relevant.*
Protocol for preconditioning of the device before its characterization	☐ Yes ☒ No	*Provide a description of the protocol.* / No preconditioning protocol
Stability of the J-V characteristic	☐ Yes ☒ No	*Provide a description of the method used. The stability of the J-V characteristic can be verified with time evolution of the maximum power point or with the photocurrent at maximum power point; see ref. 5 for details.* / We only tested the light stability in our lab.

3. Hysteresis or any other unusual behaviour

Item	Yes/No	Description / Explanation
Description of the unusual behaviour observed during the characterization	☐ Yes ☒ No	*Provide a description of hysteresis or any other unusual behaviour observed during the characterization.* / No hysteresis or other unusual behaviour was observed during the characterization of the solar cells. In general, organic solar cells do not have hysteresis problems.
Related experimental data	☐ Yes ☒ No	*Provide a description of the related experimental data.* / No hysteresis or other unusual behaviour was observed during the characterization of the solar cells.

4. Efficiency

Item	Yes/No	Description / Explanation
External quantum efficiency (EQE) or incident photons to current efficiency (IPCE)	☒ Yes ☐ No	See Fig. 3b / *Explain why this information is not reported/not relevant.*
A comparison between the integrated response under the standard reference spectrum and the response measure under the simulator	☒ Yes ☐ No	The integrated Jsc values from EQE spectrum agree well (around 3% mismatch) with the Jsc values from J-V measurement. / *Explain why this information is not reported/not relevant.*

April 2023

For tandem solar cells, the bias illumination and bias voltage used for each subcell

☐ Yes
☒ No

Provide a description of the measurement conditions.

No tandem solar cell was reported in this manuscript.

5. Calibration

Light source and reference cell or sensor used for the characterization

☒ Yes
☐ No

Enli Technology AAA solar simulator (SS-F5) and standard Si (SRC-2020) reference cell were used during solar cells testing (Methods).

Explain why this information is not reported/not relevant.

Confirmation that the reference cell was calibrated and certified

☒ Yes
☐ No

The reference cell (SRC-2020) was calibrated by NREL.

Explain why this information is not reported/not relevant.

Calculation of spectral mismatch between the reference cell and the devices under test

☐ Yes
☒ No

Provide a value of the spectral mismatch and/or a description of how it has been taken into account in the measurements.

No spectral mismatch calculation was performed in our lab.

6. Mask/aperture

Size of the mask/aperture used during testing

☒ Yes
☐ No

A 0.061 cm2 aperture was used during solar cells testing (certified 0.0608 cm2, Enli Tech. Optoelectronic Calibration Lab., Taiwan, Accreditation Criteria: ISO/IEC 17025:2017).

Explain why this information is not reported/not relevant.

Variation of the measured short-circuit current density with the mask/aperture area

☐ Yes
☒ No

Report the difference in the short-circuit current density values measured with the mask and aperture area.

We didn't measure the solar cells with apertures of different sizes.

7. Performance certification

Identity of the independent certification laboratory that confirmed the photovoltaic performance

☒ Yes
☐ No

Solar cells were certified by Enli Tech. Optoelectronic Calibration Lab., Accreditation Criteria: ISO/IEC 17025:2017

Explain why this information is not reported/not relevant.

A copy of any certificate(s)

☒ Yes
☐ No

See Supplementary Fig. 20, Supplementary Information

Explain why this information is not reported/not relevant.

8. Statistics

Number of solar cells tested

☒ Yes
☐ No

30 or 20 devices for each condition were tested.

Explain why this information is not reported/not relevant.

Statistical analysis of the device performance

☒ Yes
☐ No

See Table 2 and Supplementary Table 4

Explain why this information is not reported/not relevant.

9. Long-term stability analysis

Type of analysis, bias conditions and environmental conditions

☒ Yes
☐ No

See Supplementary Fig. 21, Supplementary Information

Explain why this information is not reported/not relevant.
